# Juice Quality Evaluation with Multisensor Systems—A Review

**DOI:** 10.3390/s23104824

**Published:** 2023-05-17

**Authors:** Emilia Osmólska, Monika Stoma, Agnieszka Starek-Wójcicka

**Affiliations:** 1 Department of Power Engineering and Transportation, Faculty of Production Engineering, University of Life Sciences in Lublin, 20-612 Lublin, Poland; emilia.osmolska@up.lublin.pl; 2 Department of Biological Bases of Food and Feed Technologies, Faculty of Production Engineering, University of Life Sciences in Lublin, 20-612 Lublin, Poland; agnieszka.starek@up.lublin.pl

**Keywords:** juice quality, food industry, sensors, multisensory systems, electronic nose, electronic tongue

## Abstract

E-nose and e-tongue are advanced technologies that allow for the fast and precise analysis of smells and flavours using special sensors. Both technologies are widely used, especially in the food industry, where they are implemented, e.g., for identifying ingredients and product quality, detecting contamination, and assessing their stability and shelf life. Therefore, the aim of this article is to provide a comprehensive review of the application of e-nose and e-tongue in various industries, focusing in particular on the use of these technologies in the fruit and vegetable juice industry. For this purpose, an analysis of research carried out worldwide over the last five years, concerning the possibility of using the considered multisensory systems to test the quality and taste and aroma profiles of juices is included. In addition, the review contains a brief characterization of these innovative devices through information such as their origin, mode of operation, types, advantages and disadvantages, challenges and perspectives, as well as the possibility of their applications in other industries besides the juice industry.

## 1. Introduction

Nowadays, access to information is easy and unlimited due to technological development. At the same time, consumers are becoming increasingly aware of the quality of the products that are being offered. Enterprises, especially those operating in the food industry, are assessed not only in terms of economics but also in terms of quality.

The entire food production process, from identifying consumer needs to launching the finished product on the market, is complex and lengthy. A lot research and analysis is required in order to understand the consumer expectations, production costs, composition, and benefits of a given product [1,2]. The biggest challenge is to ensure the appropriate quality and safety of the product, which is examined and analysed by scientists from various disciplines [3].

The parameters determining the quality of a food product include safety, nutritional value, composition (the quality of the ingredients used), and taste. Particularly noteworthy are the juices, which, if they are of high quality, should not contain preservatives, added sugar, or microorganisms that could harm the health of the purchaser. Another important parameter is the shelf life of the juice, which is inextricably linked to its packaging; it should be added that packaging primarily affects the sense of sight [4,5,6]. All these factors are interrelated and consequently affect the quality and safety of fruit and vegetable juices [7].

Juices are products made from ripe, fresh, frozen, or stored fruits and vegetables. They are obtained by pressing the juice from the pulp or by mechanically rubbing the raw materials. Depending on consumer preference, juices can be available in various forms, such as cold-pressed juices, pasteurized juices, NFC juices, etc. To extend shelf life, many juices are pasteurized to in order destroy the microorganisms and enzymes that could lead to spoilage [8].

Food products, including fruit and vegetable juices, provide many valuable nutrients that are necessary for the proper functioning of the body, such as vitamins, minerals, dietary fibre, flavonoid compounds, phytoestrogens, pectin, easily digestible sugars, and organic acids [9,10,11]. Therefore, juice producers want to avoid negative changes in taste, smell, and colour that may affect the quality and safety of food products. This problem especially applies to one-day-old juices and those that have been poorly stored. This is very important because microbiological contamination can occur already at the cultivation stage, which is when fruit and vegetables are exposed to contamination from soil, water, and air [12,13,14,15].

Despite the introduction of control measures to increase food safety, cases of food poisoning due to the consumption of juices that have been contaminated with microorganisms still occur worldwide [16,17,18]. Microorganisms capable of multiplying in juices, primarily yeasts and moulds, are the main causes of spoilage in unpasteurized and poorly stored juices, especially when the pH of the product is low. Although most of them are not harmful to humans, their presence in large quantities can lead to organoleptic changes and, in extreme cases, to complete spoilage of the product. Typical examples of undesirable flavours of substances that can appear in juices as a result of pathogenic microorganisms include odours caused by rancidity (fat oxidation), mustiness (mycotoxins), and faecal or earthy odours (geosmin and 2-methylisoborneol). In addition, typically metallic and astringent tastes may occur due to the presence of ferrous, copper, or zinc. Multisensory systems can independently detect such undesirable flavour substances in food and beverages [19,20,21,22]. According to the ECDC (European Centre for Disease Prevention and Control) and EFSA (European Food Safety Authority), juices are one of the most common sources of foodborne human illnesses [23,24,25]. Additionally, alcoholic beverages or junk food contribute to improper and unhealthy nutrition and to human illness.

To ensure the safety and the highest quality of food products, each stage of the production process is controlled, from obtaining raw materials, through processing, transport, trade, and to consumption [26]. The assessment of the quality of these products is based primarily on sensory analysis, chemical composition, physical properties, degree of microbiological and toxic contamination, as well as on the method of storage, packaging and labelling [27,28].

For many years, many different laboratory methods have been developed that allow for the qualitative and quantitative evaluation of food products. In laboratories, traditional analytical methods such as titration and weighing are used, as well as more advanced instrumental methods. Many laboratories also use modern analytical equipment. Separation techniques such as thin layer chromatography (TLC), gas chromatography (GC), and high performance liquid chromatography (HPLC) are the most commonly used methods. Depending on the purpose of the analysis and the type of sample, the chromatographs are combined with appropriate detectors, such as a mass spectrometer (MS) or a UV-Vis Spectrum [29]. Unfortunately, such devices are often very expensive. Furthermore, they require specialized staff, and the analysis can be complicated and time-consuming. For this reason, alternative methods that could allow for a quick and inexpensive analysis of samples are currently being explored.

As already mentioned, contemporary consumers are particularly interested in the quality of products through their appearance, smell, and taste, which are difficult to assess by standard laboratory methods. To overcome this problem, organoleptic tests involving a sensory panel are used. A group of trained volunteers use their senses such as sight, taste, smell, and touch to evaluate the products. However, the sense of taste is very complex and individual for each person, making the results of the research subjective [30]. In the case of products such as alcohol, cheese, juices, or coffee, whose market value depends on taste and brand, there is a need for an objective assessment of these features. In response to this, modern analytical tools have been proposed that enable for the fast and low-cost control of the production process and the quality of food products. In addition, research on electronic senses and nanotechnology contributes to the development of objective methods for evaluating sensory experiences.

These studies are conducted as part of biomimetics, i.e., an interdisciplinary field that uses the principles of engineering, chemistry, and biology to synthesize materials, synthetic systems, or machines whose functions mimic biological processes [31,32,33,34]. This field is gaining popularity as people are constantly trying to design technologies, devices, materials, etc., that closely resemble nature. Therefore, it is used in various areas, including sensory recognition, which means that it provides the possibility of digital processing using sensors of various human senses. Examples include image sensors (sense of sight), speakers (sense of hearing), pressure sensors (sense of touch) [35,36], the electronic nose (sense of smell), and the electronic tongue (sense of taste). It should be added that in many cases these sensors are much more sensitive than human sense organs.

Therefore, this article attempts to review modern multi-sensor systems, including the electronic nose and electronic tongue, for assessing the quality of fruit and vegetable juices. The article is divided into the following sections: Section 2, the essence and characteristics of the electronic nose; Section 2.1, the origin, operation, types, advantages, and disadvantages of the e-nose; Section 2.2, the application of the electronic nose in the quality analysis of fruit and vegetable juices; Section 3, the essence and characteristics of the electronic tongue; Section 3.1, origin, operation, types, advantages, and disadvantages of the e-tongue; Section 3.2, application of the electronic tongue in the quality analysis of fruit and vegetable juices; Section 4, challenges and perspectives; Section 5, the conclusions.

## 2. The Essence and Characteristics of the Electronic Nose

### 2.1. Origin, Operation, Types, Advantages, and Disadvantages of the E-Nose

Research and work on the construction of an “artificial nose” for the measurement, identification, and classification of odours has been carried out since the 1950s, when Hartman (1954) designed a simple microelectrode-based sensor for odour recognition [37]. In turn, the first attempts to create an innovative method by using a matrix of sensors were carried out already in the early 1960s. At the time, a device called a mechanical nose was developed [38]. Three years later, Wilkens and Hatman presented the electronic nose, which is a sensor array using a redox reaction on the electrode [39]. It was not until 20 years later that the concept of the first device consisting of an intelligent electronic matrix of chemical sensors was created, enabling the classification of odours [40] and their detection by using an integrated system of sensors [41]. In 1988, Gardner and Bartlett coined the term “electronic nose” to describe an instrument composed of a set of sensors capable of recognizing simple and complex odours: *“an instrument which comprises an array of electronic chemical sensors with partial specificity and appropriate pattern recognition system, capable of recognizing simple or complex odors*” [42,43].

**Figure 1:**
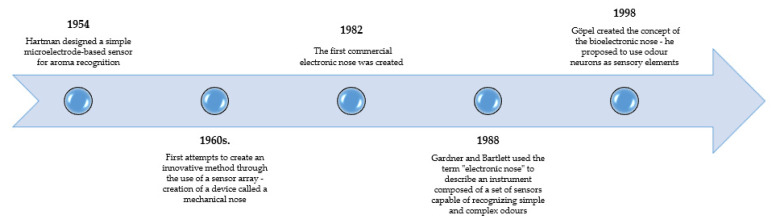
A brief history of the origins of the electronic nose, developed on the basis of Hartman, Moncrieff, Wilkens, and Gardner [37,38,39,42].

In 1982, the first commercial electronic nose was developed [40]. Then, in 1998, Göpel created the concept of a bioelectronic nose—he proposed using scent neurons as sensitive elements. He suggested that the biomolecules present on the surface of the sensors could be used to develop high-sensitivity sensors (with detection limits similar to those of dogs, for detecting drugs, explosives, etc.) [44]. Figure 1 shows a brief history of the development of the electronic nose.

Currently, the term e-nose is understood as various devices designed to imitate human olfactory perception, i.e., enabling the analysis of signals regarding the properties of a given product/mixture in a way that resembles the operation of an olfactory analyser in the brain [43,45,46,47,48]. Thus, it can be simplified to say that the e-nose is a system analogous to the sense of smell and that the sensors play the role of the olfactory epithelium because the mechanisms for identifying odorous substances implemented in electronic devices are very similar to those in the human nose [49].

The human sense of smell is quite complex, as humans have the ability to perceive thousands of different smells—the olfactory senses can distinguish 10,000 types of smells using approximately 400 types of receptors [50,51]. This is determined by the complex structure of the olfactory epithelium located in the nasal cavity. Several million olfactory neurons are located there, and at the ends of their modified dendrites there are hair-like extensions that detect odorous substances. Due to the spatial segregation of neurons and axons, each odourant stimulates a specific set of olfactory glomeruli.

The principle of operation of the e-nose is that the sensor system selectively reacts to odorous compounds or their groups contained in the tested sample—that is, it determines the odour consisting of a large amount of various volatile substances in the space above the sample, and then provides output data that represent the “fingerprint” of all sample components [52]. This means that the “smell” is recognized when the analysed set of signals is sufficiently similar to the analogous set corresponding to the “pattern”.

Depending on the application of the system, different methods are used to construct the patterns and determine the degree of compatibility of both sets. However, an e-nose always consists of a set of sensors, consisting of several or several dozen elements, i.e., a multi-sensor matrix, an information processing unit, such as an artificial neural network (ANN), software with digital pattern recognition algorithms, and databases [53,54]. It is worth mentioning that these elements in commercial devices are mounted in easily replaceable modules, allowing the device to be adapted to the task performed.

Chemometric methods based on the use of one of many analytical techniques such as Principal Component Analysis (PCA), Discriminant Analysis (DA), Discriminant Function Analysis (DFA), Support Vector Machine (SVM), Partial Least Squares (PLS), Kernel Principal Component Analysis (KPCA), and single and multivariate Analysis of Variance (ANOVA) can be used to interpret the results obtained with the electronic nose. Artificial Neural Networks (ANN) are also used here [55,56,57,58,59].

Most often, electronic noses are constructed on the basis of conductometric sensors, which use the changes in the electrical conductivity of the active material, for example metal oxides (MOS) or some polymers, as well as piezoelectric sensors (quartz microbalances) and biosensors [49]. However, broadly speaking, the sensors used to build the matrix of electronic noses can be divided into several classes due to their construction and principle of operation [60,61,62,63]:

- Electrochemical (change in potential or resistance caused by charge transfer);
- Thermal (temperature change caused by chemical action);
- Mass (mass change due to the absorption of a substance);
- Optical (changes in light intensity).

Chemical sensors most often work on the following principles: 1. Change of resistance: MOS (metal oxide semiconductor), CP (conducting polymer), polymer composite, 2. Change of potential: MOSFET (metal oxide semiconductor field effect transistor), 3. Frequency changes: BAW (bulk acoustic wave), SAW (surface acoustic wave), 4. Other, e.g., mass spectrometer. Attention should also be paid to biosensors in which the active element is a biomaterial deposited on a suitable transducer.

Artificial noses are currently used as a supplement to various methods of odour analysis, as they eliminate the various imperfections of classical methods (GC/MS, sensory panels, trained animals) such as, e.g., the subjectivity of sensory panels, analysis of substances at high concentration levels or the need to employ specialized personnel, long analysis time, and high prices.

Electronic noses are an objective, automated non-destructive technique characterized by their high sensitivity to chemicals (which is comparable to the sensitivity of the human nose), ease of construction, and cost-effectiveness [64]. In addition, the use of this multi-sensor system allows for faster and cost-effective odour analysis.

Among the other advantages of this measurement system, the following can also be mentioned [65,66,67,68]:

- High resistance to changing weather conditions, mainly temperature and humidity,
- “medium selectivity” in relation to “foreign” substances present in the gas mixture, which is sometimes more flavourful;
- High stability, repeatability, and reproducibility;
- Short response and recovery time;
- Easy calibration and a simplified data processing system;
- The device is often small in size.

It should also be added that many of the used sensors can improve the measurement capability of the system. However, on the other hand, it should also be remembered that the use of a large set of sensors results in a greater computational effort and time required to process the obtained data. In addition, there may be redundant sensors in multi-component systems, i.e., sensors that do not generate significant signals. Another disadvantage of the analysed measurement method is the limited scope of its application [69,70].

Table 1 below summarises the main advantages and disadvantages with a brief explanation.

Despite the disadvantages mentioned above, it seems that further development of odour analysis is related to the use of electronic noses. It should be added that they are constantly being improved, especially in the direction of increasing their sensitivity, specificity, selectivity, accelerating the response time, while simplifying the principles of operation, and consequently their operation, as well as their miniaturization and increasing their mobility [71].

**Figure 2:**
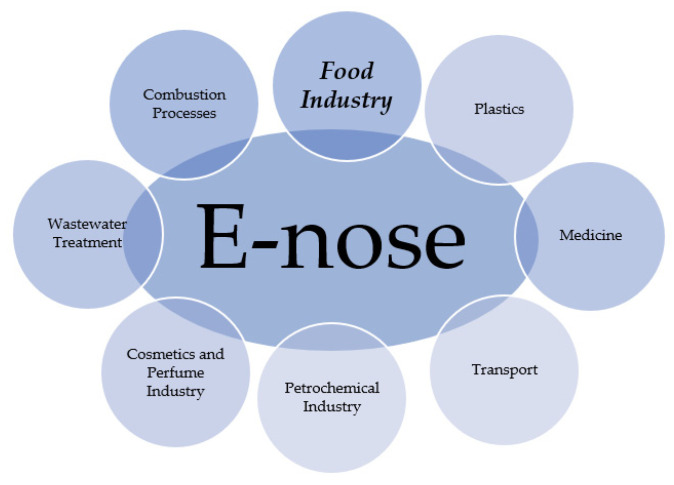
E-nose application areas, developed on the basis of Krzyżewska and Pawlak-Lemańska [89,90].

Electronic noses are currently quite widely used in many different market branches, as shown in Figure 2. They can be used in medical research (e.g., in the detection of disease biomarkers, selection of therapy, metabolic disorders or organ dysfunctions, detection and recognition of eye bacteria or bacteria in the blood, detection of blood in the urine, etc.) [72,73,74,75,76,77,78], forensic and criminology [79,80], and in the cosmetics industry, in particular perfumery (e.g., for authenticity assessment, for the development of new fragrances) [81,82], pharmaceutical industry (for quality control, purity and homogeneity of drug composition, and product safety) [70], and food industry (e.g., for testing compliance with standards, marking product composition, getting to know the brand, marking impairment on the shelf, etc.) [60,83,84], as well as in agriculture (for assessing the maturity of crops, determining the date of harvest and storage time, assessing the degree of contamination, characterizing varieties, and diagnosing plant diseases) [69,85,86,87,88].

Electronic noses are also useful in monitoring and environmental protection, e.g., during the measurement of the degree of contamination of environmental samples (water, air), the emission of unpleasant and harmful volatile substances or poisonous leaks, and the assessment of the effectiveness of wastewater and waste gas treatment, or the assessment of their odour nuisance [91,92,93,94,95,96]. As an example, research conducted in Poland can be cited here. Since 2021, the SENSODOR project has been implemented at the Wrocław University of Technology. As part of this project, an e-nose is being developed to help detect sources of nuisance odours and monitor their levels in the environment. The implementation of the project is expected to take three years. The final stage of the project, after developing an electronic nose and creating a monitoring network, will be installing it on a drone. This type of autonomous device could therefore be used, among others, in to search for sources of odour nuisances [97].

Electronic noses are also used to analyse fuel components [98] and test groundwater quality [99]. They are also used in the military industry (e.g., for identifying combat gases, detecting mines, and testing the air quality inside vehicles, e.g., space vehicles) [71], as well as for detecting hazardous substances, including flammable and explosive substances (e.g., at airports) [100]. They are also used in the monitoring of production processes and in scientific research in various fields.

### 2.2. Application of the Electronic Nose in the Quality Analysis of Fruit and Vegetable Juices

As mentioned earlier, the use of electronic noses in the food industry is particularly widespread. They are applied in many different areas, such as food quality control [101,102], e.g., shelf life testing [103,104,105], freshness assessment [106,107,108,109,110,111,112], processing monitoring [83,113,114,115], monitoring of storage processes [116], and assessment of authenticity [117,118,119,120]. In addition, they can be used to control the quality of production processes, monitor fermentation processes, control the quality (tightness) of packaging [121,122], or to identify products or their ingredients [123,124,125,126,127,128].

**Figure 3:**
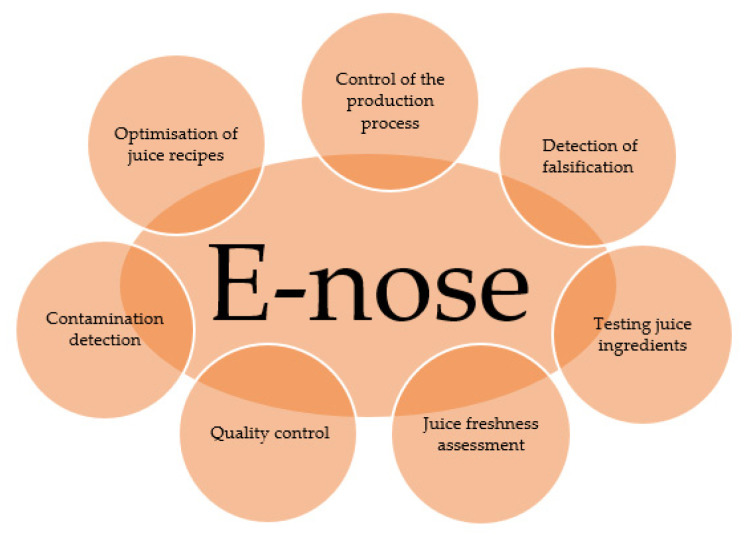
E-nose application areas in the juice sector, developed on the basis of Wahia and Li [114,130].

In the juice sector, the electronic nose can be used to identify the various aromas and flavours that are characteristic of a particular beverage. Thanks to its high sensitivity, it can be very useful in assessing the quality of the raw materials used in juice production. In addition, the electronic nose is used to check the quality of an already packaged product where shelf life problems may have occurred as a result of a poorly conducted fixing process [114,129]. Figure 3 shows the application of the electronic nose in the juice sector.

Many researchers have carried out various experiments on electronic noses. For example, Rasekh and Karami [131] used the e-nose in combination with an artificial neural network to detect the ripple effects in fruit juices by discriminating between them. They used unit-radius radar excursions to compare the patterns (i.e., the fingerprints) of juice samples. The authors of this study obtained the highest response for the sensors named MQ135 and TGS813, and the lowest response for the sensors named MQ3-4-8-9 and 136. They concluded that the selection of sensors with good discriminating characteristics could improve the classification ability. In addition, nine MOS gas sensors were used in an electronic nose. The signals from the sensors were analysed by PCR and the most suitable ones were selected. It was found that the selected sensors were effective in the qualitative evaluation of different fruit juices and could be used in the construction of an optimal machine olfaction system. The correlation between measurements and predicted fruit juice flavour parameters showed a high prediction performance based on the e-nose signals. The results suggest that e-nose in combination with ANN can be used to analyse products for authenticity with satisfactory results.

In turn, Zhang et al. [132] focused on evaluating the aroma of freshly squeezed strawberry juice stored in cold storage using e-nose, headspace solid phase micro extraction-gas chromatography-mass spectrometry (HS-SPME-GC-MS) and gas chromatography-ion mobility spectrometry (GC-IMS). Although strawberries of the ‘Hong Yan’ (HY) and ‘Xiao Bai’ (XB) species are popular in Jiangsu Province, China, the aroma profile of freshly squeezed juice from these fruits is not well understood. Electronic nose, HS-SPME-GC-MS, and GC-IMS were used to analyse the aroma of HY and XB strawberry juices stored at 4 ± 1 °C. In the beverage freshly prepared by HS-SPME-GC-MS HY and XB, 17 and 21 volatile organic compounds (VOCs) were detected, while up to 50 VOCs were detected by GC-IMS. Characteristic compounds identified included hexanal, methylpropanal, 5-methylfurfural and 2-furfural in HY, and hexyl acetate, ethyl 3-methylbutyrate, propyl hexanoate, and octanal in XB. In addition, the quality and taste of HY and XB strawberry juices were compared. The vitamin C content of HY juice was higher than in XB, but other characteristics, such as taste and colour, were less satisfactory than in XB. Aroma profiles were different between HY and XB, which was related to cultivars and chemical composition. The use of e-nose, HS-SPME-GC-MS, and GC-IMS allows for a complementary analysis of freshly squeezed strawberry juice during cold storage.

Other researchers Wang et al. [133] have focused on evaluating the aroma characteristics of sugarcane (*Saccharum officinarum* L.) juice using gas chromatography-mass spectrometry and e-nose. Juices from four sugarcane cultivars were analysed using gas chromatography-mass spectrometry (GC-MS) and e-nose to investigate the aroma characteristics. Thirteen volatile compounds were detected, including aldehydes, alcohols, phenols, and ketones. Cluster and PCA analysis of e-nose data showed that bamboo cane (BC) and yellow rind sugarcane (YS) juices formed one group, while black rind sugarcane (BS) and green rind sugarcane (GS) formed the other group. E-nose with linear discriminant analysis (LDA) allowed the sugarcane juices to be clearly distinguished. Hexane, 1-pentanol, 2-butanol, 1-penten-3-one, 2-octanol, and acetaldehyde showed high correlation with e-nose response signals. Sensory analysis showed noticeable differences in aroma between BC and GS (*p* < 0.01) and YS and GS (*p* < 0.05). The use of GC-MS in combination with e-nose can serve as an alternative tool for assessing and differentiating sugarcane juices from different varieties, due to its high sensitivity and objectivity.

Wahia et al. [130] analysed the effect of mild thermosonication (MTS) on the quality of orange juice (OJ) infused with *Alicyclobacillus acidoterrestris* (AAT) during 24-day storage using various temperatures. Changes were related to bioactive compound content, antioxidant activity, and pectin methyl esterase (PME) concentration. No differences were observed in pH and total soluble solids levels. To analyse the nutritional and microbiological properties of the juice, non-linear kinetic curves, and four-parameter models of logistic decomposition were used. The electronic nose successfully distinguished between the control and OJ-treated odour through linear discriminant analysis (LDA). Terpenes, alcohol, and partially aromatic compounds were the main indicators of OJ spoilage. The combination of e-nose with GC/MS may be an alternative to traditional food analysis techniques for the rapid detection of the microorganisms responsible for juice spoilage.

As there are many studies on the use of the electronic nose in assessing the quality of fruit and vegetable juices, Table 2 below provides examples of studies carried out over the last five years.

In summary, the electronic nose is a device capable of analysing and identifying odours, which is widely used in the food industry. Thanks to its various sensors, the e-nose can determine the chemical composition, detect contaminants and control the microbiological status of juices without the need for complex laboratory methods [124,132,147,148]. In this way the electronic nose supports quality control processes and helps ensure food safety, which results in a reduction in food waste and financial losses for processing plants and, above all, consumer satisfaction [149,150].

## 3. The Essence and Characteristics of the Electronic Tongue

### 3.1. Origin, Operation, Types, Advantages, and Disadvantages of the E-Tongue

A few years later after the appearance of the e-nose, the concept of a similar analytical tool for analysing liquid samples, the so-called Taste Sensor, was developed. The first taste sensor, or electronic tongue, was designed in the late 1980s—it was created by a Japanese scientist Susumu Kurihara in 1989. Kurihara and his team at Osaka University developed a lipid membrane taste sensor that was capable of distinguishing between five basic tastes: sweet, sour, salty, bitter, and umami. This marked the beginning of a new era in the field of taste sensing technology, paving the way for the development of more advanced electronic tongues [151].

The Hayashi Taste Sensor is an electronic tongue developed by Dr. Kiyoshi Toko and his team at the Kyushu University, Japan, in the early 1990s. Dr. Toko worked with Dr. Hidekazu Hayashi, another pioneer in the field of electronic tongues. The Hayashi Taste Sensor was designed to mimic human taste sensation and analyse the taste profile of various substances, including food and beverages [152,153].

In 1997, a significant advancement in electronic tongue technology was achieved through a collaboration between researchers from the University of Rome Tor Vergata in Italy and St. Petersburg State University in Russia. This innovative electronic tongue was developed under the guidance of Prof. Yuri Vlasov, Prof. Anatoly Legin, and Prof. Andrey Rudnitskaya. The 1997 electronic tongue utilized potentiometric sensors, specifically ion-selective electrodes, to analyse and differentiate the taste profiles of various liquid samples. The acquired data was then processed using multivariate data analysis techniques, which enabled the classification and identification of complex taste patterns. The 1997 electronic tongue marked an important milestone in the development of taste sensing technology. It demonstrated the potential of electronic tongues for various applications in the food and beverage industry, environmental monitoring, and medical diagnostics. Since then, electronic tongue technology has continued to evolve, incorporating new sensing techniques and materials, and expanding its range of applications [154,155].

Since then, many of these devices have been developed with various gas sensors [156]. The electronic tongue was equipped with potentiometric sensors so as to identify liquid samples.

**Figure 4:**
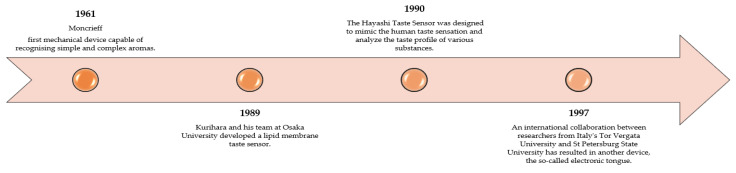
A brief history of the origins of electronic tongue, developed on the basis of Labanska, Toko, and Ciosek [29,154,155].

The development of electronic tongue technology also included the introduction of new detection methods such as electrochemical, optical, and acoustic sensors. In the following years, various electronic tongue systems have been successfully applied in food analysis, medicine, environmental protection, and pharmaceuticals [56,157,158]. Figure 4 shows a condensed history of the emergence of the electronic tongue.

The advancement in the evolution of sense organs, such as the nose and tongue, is made possible by the occurrence of biological impulse responses in mammals. The sense of smell works through a number of receptors that respond to various chemical compounds. These signals are then delivered by the nervous system to the brain, where a network of neurons configures them into response patterns. It should be mentioned that human taste testing will be irresolutely linked to long coordination, execution, and interpretation times, resulting in high costs. These concerns sometimes limit the taste evaluation in the early stages of product development. This is a very important step in designing the right organoleptic sensation. Researchers are therefore constantly looking for new solutions in this area [29,158]

The electronic tongue is an innovative analytical device that simulates the human sense of taste to identify, distinguish, and classify chemicals and liquid mixtures based on their taste characteristics [159].

**Figure 5:**
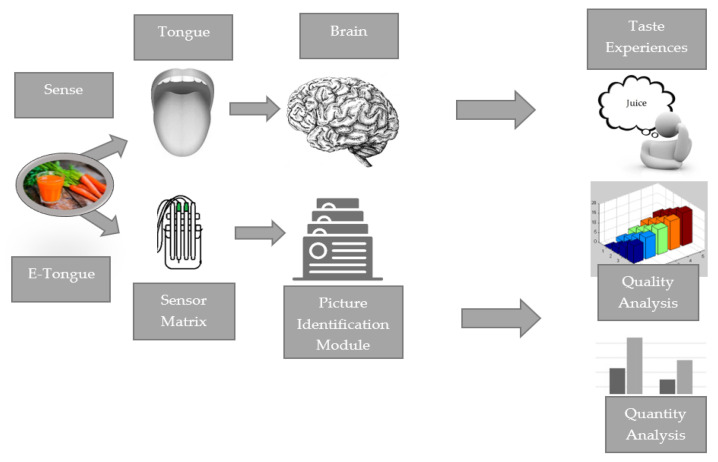
A simplified diagram of how a tongue and e-tongue work developed on the basis of Labanska [29].

A typical electronic tongue consists of several separate sensors, each used to detect specific chemical compounds or groups of compounds. These sensors work together to generate a profile of the sample that is being tested. Electronic tongues work by collecting data from sensors [157,159,160,161]. This data can then be analysed using statistical or machine learning techniques to identify and classify a sample based on its taste characteristics. In this way, an e-tongue can process information from multiple sensors to detect the subtle differences in sample composition and extract characteristic patterns, allowing samples to be classified [152,162]. Figure 5 shows a simplified diagram of how a tongue and e-tongue work.

Overall, the e-tongue is a valuable tool for analysing the chemical composition of liquid and solution samples and, similar to the human tongue, it performs a comprehensive analysis of their chemistry. The e-tongue reflects three levels of biological taste recognition: the receptor level (the taste buds in humans or probe membranes in e-tongue), the circuital level (the neural transmission in humans or transducer in e-tongue), and the perceptual level (the cognition in the thalamus in humans or computer and statistical analysis in the e-tongue) [163].

As electronic tongues are very complex devices, they consist of different types of sensors. Electronic tongues can be divided into various types depending on the sensing technologies they use [162,164]. Common types of e-tongues are listed and characterised below [165,166]:

1. Electrochemical e-tongues: They use electrodes, such as ion-selective electrodes, to measure changes in the potential in liquid samples. They are used to assess the quality of beverages such as juices, mineral water, or alcoholic beverages, as well as to analyse the composition of food;
2. Optical e-tongues: Based on fibre-optic technology, this type of e-tongue analyses changes in the light passing through a sample to obtain information about its composition. They are used in assessing the quality of food products, such as milk, honey or vegetable oils, and to detect impurities and adulteration;
3. E-tongues based on biosensor technology: They use biological molecules, such as enzymes or antibodies, to identify specific chemicals in liquid samples. They can be used to identify allergens, bacteria, or toxins in food, which is key to ensuring food safety;
4. E-tongues with mass sensors: They use mass spectrometry technology to analyse the chemical composition of liquid samples. They are used to identify flavour and aroma components in the food industry, for example in assessing the quality of coffee or tea.

The e-tongue is a system that uses an automatic liquid sampler, via a sensor array module. The e-tongue can take samples without much preparation and needs only 2–3 min to analyse. The e-tongue system consists of a probe, designed to measure dissolved compounds in samples. Each of the probe’s sensors has its own characteristic response span that interacts with other taste attributes. The integration of processes from each sensor generates a unique ‘character’. The analytical part of the system works on a matrix of sensors that are able to detect the chemical compounds in samples [114,167,168].

A large number of detection sensors are similar to or even more precise than the human sense. The human tongue and electronic tongue have different taste detection limits. For example, the detection level of sucrose (sweetness) is 1 × 10^−2^ M and 2 × 10^−6^ M for a human tongue and an electronic tongue, respectively, and the detection threshold of caffeine (bitterness) is 0.7 × 10^−3^ and 1 × 10^−6^ for the same languages, respectively. Electronic tongues have the ability to pick up chemical changes before they are noticed by human receptors. The sensor array can be adapted as required. Very importantly, measurements are made by potentiometry, and the results are recorded with reference to an Ag/AgCl reference electrode. Each probe is cross-selective, allowing for complete coverage of the taste profile. Taste recognition is done at the perceptual level, rather than in the probe, through the use of a computer. The entire instrument includes a software package that comes complete with a system containing a sophisticated chemometric setup for measurement and data analysis [163,169,170].

An electronic tongue allows sensor data to be collated with taste patterns. The results obtained are compared quantitatively, taking into account analytical parameters such as concentration or flavour intensity on a scale. Depending on the intention of the analysis being performed, the study can provide a wide variety of information. If there are new chemical compounds for which there are no achievable data that do not explicitly mention a specific taste, a useful tool is to quantify the bitterness as a function of known bitterness factors, using discriminant factor analysis (DFA). Using this method, the bitterness of the API can be compared to the bitterness of urea of a known concentration, providing a starting point for flavour optimisation. Such a solution can help in the creation of bitter placebos for blinded clinical trials [159,163,171].

With advances in technology, electronic tongues have the potential to be used in a variety of industries, which can bring many benefits to humanity and the environment [152,172,173]. Electronic tongues are constantly being developed and refined, allowing for more and more accurate and rapid detection of different substances. Similar to any device, an electronic tongue will have advantages and disadvantages [174,175]. Table 3 below shows the main advantages and disadvantages of this innovative device.

**Figure 6:**
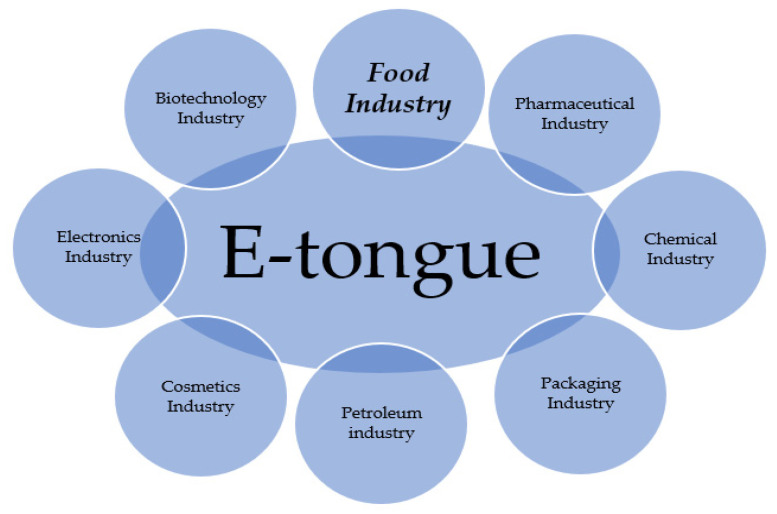
E-tongue application areas, developed on the basis of Wróblewski and Labanska [29,177].

It is worth mentioning that these are not all the advantages as well as disadvantages that may arise during the operation of such a device. In addition, it should be noted that, although electronic tongues have many advantages, there may also be limitations, such as difficulties in identifying individual substances in mixtures or the limited lifetime of some sensors. Nevertheless, electronic tongues are becoming increasingly sophisticated and are widely used in many areas [178,179,180,181,182], such as food and beverage analysis, environmental monitoring, and medical diagnostics. In food research, e-tongues have been used to detect contaminants, adulteration, and quality control. In environmental protection, e-tongues allowed for the identification of toxins and other harmful substances. In medicine, e-tongues were used to diagnose diseases, monitor patients, and detect biomarkers in body fluids [56,159,183]. In addition, e-tongues can be used in the cosmetics, fuel, electronics, and biotechnology industries [52,139,153,154]. Overall, the development of e-tongues has provided a valuable tool for analysing and understanding chemical composition. Figure 6 shows in which industries the e-tongue can be used.

Various models are currently available on the market, including the popular PEN manufactured by German company Airsense Analytics, the FOX models, and the latest Heracles offered by French company AlphaMOS [184]. Electronic tongues have been used, among other things, in the control of food production processes [185], the detection of human and plant diseases [43,56], and to identify the possible contamination of food products by pathogens [186].

In summary, electronic tongues are a valuable tool for understanding and analysing the chemical composition of liquids and solutions, with significant potential for future research and applications.

### 3.2. Application of the Electronic Tongue in the Quality Analysis of Fruit and Vegetable Juices

Particular emphasis should be placed on its application in the food industry due to its ability to analyse and assess the taste, chemical composition, and quality of food products. Very importantly, the food industry is a particularly difficult sector due to perishable products such as juices, dairy, and meat. In the food industry, e-tongues are widely used in various areas, such as:

1. Quality control: Monitoring the quality of raw materials, intermediate and final products, as well as detecting contamination and falsifications;
2. New product testing: E-tongues can help develop new recipes and flavours, as well as assessing the impact of changing ingredients on the taste and quality of products;
3. Food safety: E-tongues can be used to detect the presence of bacteria, toxins, or allergens in food products, which is key to protecting consumer health;
4. Optimisation of production processes: E-tongues can be used to provide real-time monitoring of production processes [114,168,172,176].

The use of electronic tongues is so extensive that only a few possible applications have been outlined. The most important is the fact that electronic tongues are able to detect contamination and adulteration of food products, so that the quality and safety of the food remain at a high level. This is particularly important for consumers, who are becoming increasingly aware and concerned about their health [187,188].

**Figure 7:**
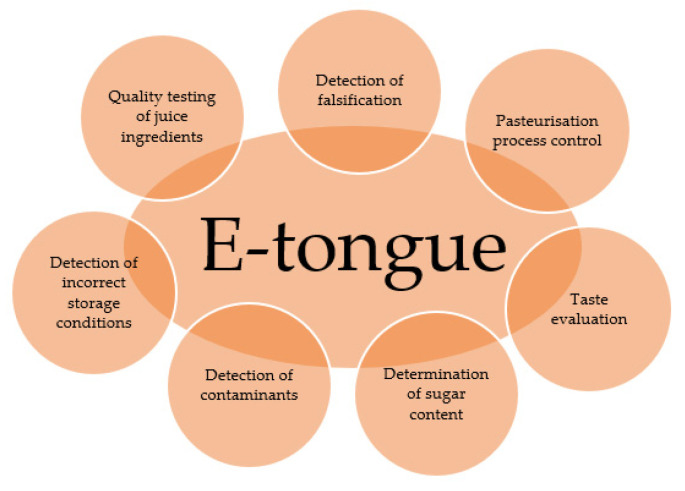
E-tongue application areas in the juice sector, developed on the basis of Wadehra and Legin [174,176].

As mentioned earlier, e-tongues have a wide range of applications, especially in the food industry, where they are used to test the quality of various products such as wine, beer, coffee, or juices. They are used to test the composition of products, to search for and identify flavours, to compare competing products or to test the effect of storage conditions on the shelf life of final products. Figure 7 shows the use of electronic tongue in the juice sector.

For example, Hong and Wang [189] used an e-nose and e-tongue to measure the freshness of cherry tomatoes for juice production. The study showed that the freshness of these fruits can be measured using sensor systems such as an e-nose and e-tongue. The authors of the study found that the e-tongue had better prediction performance than the e-nose, with higher quadratic correlation coefficients and lower standard error of prediction. The study was conducted at different storage temperatures (4 and 25° Celsius) and durations of shelf life (SLs, 16 days at 4° Celsius and 8 days at 25° Celsius). Quality indicators of cherry tomatoes are SLs, pH, soluble solids content (SSC), vitamin C concentration (VC), and firmness. In general, it was found that it is possible to determine the freshness of fruit destined for juice using sensor systems and that one tool may be sufficient, without the need to combine them.

Qiu et al. [190] used an e-tongue and e-nose to characterise five types of freshly squeezed strawberry juices, as well as juices treated to microwave pasteurisation, steam blanching, short-term high-temperature pasteurisation, freezing, and then defrosting. Juice quality parameters (vitamin C, pH, acidity, and total soluble solids) were determined by traditional measurement methods. Qualitative classification and quantitative regression were performed using multivariate statistical methods (linear discriminant analysis (LDA) and partial least squares regression (PLSR)) and neural networks (Random Forest (RF) and Support Vector Machines). The e-tongue system achieved higher accuracy rates than the e-nose, and simultaneous use had an advantage in LDA classification and PLSR regression. According to the cross-validation, RF showed excellent and indisputable results in qualitative and quantitative analysis. This work indicates that the simultaneous use of an e-nose and e-tongue can be a useful technique for detecting the degree and/or manner of juice processing.

The aim of the study of Vitalis’a et al. [159] was to use advanced analytical techniques such as near-infrared spectroscopy (NIRS) and electronic tongue to detect *M. fructigena* fungal infection on plums and to quantify this contaminant in the juices pressed from them. The plums were inoculated with the fungus in different ways and stored under different conditions, and the results obtained with both apparatuses were analysed using chemometric methods. The NIRS-based method proved successful in detecting infection before visible signs of spoilage appeared, while the e-tongue was able to quantify the level of these contaminants. Both methods proved suitable for discriminating treatment groups, but the classification accuracy was higher for samples stored at 24 °C. The results of the study indicate that both NIRS and e-tongue can be effective tools in reducing food waste through the rapid determination of fruit quality.

Moreover, Wang and Sun [191] investigated the potential use of an electronic tongue in combination with chemometric analysis for the early detection of *Zygosaccharomyces rouxii* in apple juice. The study used an electronic tongue to identify this contamination, and relied on a taste evaluation by a panel of tasters. Through discriminant analysis, contaminated juice was identified after 12 h when the yeast population was less than 2.0 log10 CFU/mL. At this level, the panellists did not identify spoilage. The HA, ZZ, BB, and BA sensors were sensitive to changes in juice taste. The number of *Z. rouxii* cells could be determined by regression models with a high coefficient of determination of 0.98–0.99. The electronic tongue proved effective in detecting this yeast species at an early stage.

Cai et al. [137] investigated the effect of different strains of lactic acid bacteria on the flavour profile of fermented jujube juice. The researchers used an electronic nose and tongue to assess the effect of LAB on the taste of the juices. They observed differences between beverages fermented with different probiotic strains. They found that jujube juice fermented by *L. plantarum* had more aromatic compounds and less volatile sulfur compounds. The taste of the juice containing the *L. plantarum* strain had adequate acidity, which was preferred by consumers.

As there are many different studies on the use of an electronic tongue in evaluating the quality of fruit and vegetable juices, Table 4 below provides examples of studies carried out in the last five years. It should be added that the table includes fewer examples of e-tongue application in the juice industry than was the case with e-nose (Table 2), due to the fact that slightly fewer studies have been completed for e-tongue.

In conclusion, today’s electronic tongues are becoming increasingly sophisticated, allowing them to be widely used in the juice industry for the benefit of both producers and consumers.

## 4. Challenges and Perspectives

Alongside the many potential opportunities for electronic tongues and noses, they will also face various challenges that will affect their further development and widespread use [68,203]. To sum up the topics related to the use of these devices, the prospects and potential challenges can be mentioned:

− Technological scale: improving the technologies used in e-tongues and e-noses is key to increasing their sensitivity, selectivity, and speed. Innovations in nanotechnology, materials or biotechnology can contribute to the development of new, more advanced sensors [29];
− Integration with other systems—e-tongues and e-noses have greater potential when integrated with other measurement systems, such as image analysis or chemical systems. This multi-parametric approach can lead to a better understanding of complex samples and more precise results [19];
− Portability and miniaturization—the development of portable and miniaturised e-tongues and e-noses devices will be key to their widespread use, especially in environmental monitoring or on-site medical diagnostics [21];
− Artificial intelligence and machine learning—the use of advanced data analysis techniques, such as artificial intelligence or machine learning, can help to improve the interpretation of measurement results, identify patterns, and speed up the decision-making process [56];
− Standards and regulations—the introduction and harmonisation of standards and regulations for e-tongues and e-noses can contribute to greater confidence in these technologies and their widespread use in various fields, such as the food industry, medicine, or environmental protection [27];
− Cost—reducing the cost of producing and using e-tongues and e-noses will be important for their wider use, especially in sectors with limited financial resources [92];
− Education and training—introducing training and education programs related to e-tongues and e-noses can help to improve potential users’ understanding of these technologies and contribute to their widespread adoption [29];
− Interdisciplinary collaboration—collaboration between scientists, engineers, doctors, and entrepreneurs from different fields can contribute to the further development and innovation in the use of e-tongues and e-noses [29].

These are just a few of the challenges that arise from the development of these advanced devices.

## 5. Conclusions

Innovative methods to analyse samples faster, more efficiently, and more cost-effectively have long been sought after. Electronic tongues and noses are the kind of devices that can provide as much information as possible about a sample with a single test in an easy way. They are an alternative or complement to complex and costly classical analytical techniques, allowing for the direct examination of samples without the need for prior preparation.

Moreover, they enable the solution of complex research problems that do not depend on just one characteristic of the sample, such as the confirmation of wine authenticity. It should be noted that the best results are achieved when these two devices are used together, as their combination gives the best results.

However, although all the applications presented in this article seem promising for fruit and vegetable juice analysis, electronic tongues and noses are still in the early stages of development and need more time to be widely implemented. Therefore, the subject matter is so interesting and extensive that it is worth exploring through such reviews.

## Figures and Tables

**Table 1:** The main advantages and disadvantages of an electronic nose [19,21,43,47,53,56,65].

Advantages		Disadvantages	
Advantage	Brief Explanation	Disadvantage	Brief Explanation
Detection of odours [65].	The electronic nose can detect a variety of odours that the human nose may miss or not notice. It can be used to detect unpleasant odours, as well as chemicals, gases, or even odours of criminal substances [65].	Limited ability to detect subtle odours [19].	Electronic noses can have difficulty detecting very subtle odours that the human nose can sense. They can miss subtle nuances and details, which can affect the precision of the analysis [19].
Speed and precision [19].	The electronic nose can detect and analyse odours in a very short time. This allows specific odours to be identified immediately, which is particularly useful in time-sensitive situations [19].	Need for calibration and maintenance [56].	Electronic noses require regular calibration and maintenance to maintain their accuracy and effectiveness. This can be a time-consuming and costly task, especially for large systems or sensor networks [56].
Multi-tasking [21].	The electronic nose can be used to identify different odours simultaneously. It can detect and distinguish between different chemicals or odours in the same room, which is not possible with a regular nose [21].	Sensitivity to external conditions [47].	Electronic noses can be sensitive to changes in temperature, humidity, as well as to the presence of other substances in the environment. Extreme weather conditions or contamination can affect their performance and accuracy [47].
Convenience of use [43].	The electronic nose can be carried and used in a variety of places and situations. It can be used in industry, emergency services, medicine, or even in everyday applications [43].	Cost [65].	Electronic noses, especially those using advanced technology, can be expensive to both purchase and maintain. Some may be too expensive for small businesses or organisations [65].
Objectivity [46].	The electronic nose works on the principle of sensors and analyses odours objectively, regardless of biases or personal preferences. This provides more objective results and eliminates individual differences in odour perception [46].	Complexity of data analysis [19].	Data collected by electronic noses can be complex to analyse. It requires sophisticated algorithms and data processing techniques to properly interpret and use the information associated with the odours [19].
Remote monitoring capability [47].	The electronic nose can be remotely monitored and controlled. Data on detected odours can be transmitted to the relevant persons or systems, allowing for a rapid response in the event that dangerous or abnormal odours are detected [47].	Lack of versatility [46].	Electronic noses may be optimised to detect specific groups of odours or chemicals. Some models may not be able to detect a wide range of different odours simultaneously, limiting their use in certain industries [46].
Time and cost savings [65].	An electronic nose can speed up the inspection and diagnostic processes by eliminating the need for manual sample searching or testing. This saves time and costs associated with traditional odour detection methods [65].	Complicated calibrations for different odours [43].	If an electronic nose is to be used to detect different odours, it may require complex calibration processes for each substance or odour group. This can be time-consuming and require the involvement of specialists [43].
Safety [19].	An electronic nose can help identify hazardous chemicals, gases, or odours that may pose a health or safety risk. It can be used to detect spills or contaminants in the environment, chemical industries, refineries, manufacturing plants, etc. In this way, appropriate countermeasures can be taken quickly and potential risks to workers and the environment can be minimized [19].	Risk of false results [21].	Electronic noses can be prone to erroneous readings or false results. Factors such as interference from other odours, contamination, or sensor damage can lead to incorrect results, challenging the accuracy of the analysis.
Innovation and technological development [21].	The electronic nose is an example of advanced technology that is developing and evolving. Research in this area is leading to continuous improvements in odour sensors, which can have a positive impact on many fields, from science and medicine to industry and public safety [21].	Dependence on programming and algorithms [56].	Electronic noses operate on the basis of programming and algorithms that determine what odours are to be detected and how they are to be interpreted. Incorrect programming or insufficiently precise algorithms can lead to incorrect results or inaccurate analysis [56].
Research and analysis of smell quality [43].	The electronic nose can be used for research and analysis of odour quality in various industries. It can help assess the quality of food products, cosmetics, perfumes, wines, coffees, etc. By accurately determining the fragrance ingredients and their proportions. This enables manufacturers to improve products and provide a better fragrance experience for consumers [43].	Lack of ability to subjectively assess odours [19].	Electronic noses do not have the ability to subjectively assess odours like humans. They are not able to sense context, emotion, or individual preferences, which can be important in certain fields such as the perfume industry or gastronomy [19].

**Table 2:** Examples of studies carried out over the last five years using e-nose in assessing the quality of fruit and vegetable juices.

Authors, Publication Year	Type of Juice Analysed	Object of Research	Selected Results
Liu et al. (2018) [134]	Watermelon juice	Comparison of fresh juice aroma characteristics of different varieties; identification and quantification of the fresh watermelon juice aroma of five varieties.	In watermelon juice, 55 volatile substances were identified, including 6 volatile substances identified for the first time in watermelon.
Wang et al. (2019) [133]	Sugarcane (*Saccharum officinarum* L.) juice	Investigating the properties of aroma, assessing the ability to differentiate juices.	Thirteen volatile compounds were identified. Alcohols were identified as the main volatile components in all sugarcane juice. It was concluded that e-nose in combination with gas chromatography-mass spectrometry (GC-MS) can be used as an alternative approach to evaluate and distinguish sugarcane juice produced from different varieties because of its high sensitivity and objectivity.
Cao et al. (2019) [135]	Citrus juice	Classification of citrus juice from different days of storage.	An improved label consistent KSVD (L-KSVD) method called E-LCKSVD for e-nose has been proposed, which can improve its performance.
Niu et al. (2019) [136]	Apple juice	Characterisation of apple juice ester odourants quantitative measurements, odour threshold, and aroma intensity analysis.	Perceptual interactions between key apple juice esters (identified and selected by GC-O, GC-MS, OAVs of compounds, and omission experiments) were investigated using odour thresholds, aroma intensity, and e-nose analysis. It was shown that fruity, sour, and “green” note attributes covaried well with the five sensors of the e-nose.
Cai et al. (2019) [137]	Jujube juice	Investigating the effect of different Lactic Acid Bacteria (LAB) strains on the flavour profile of fermented jujube juice.	A clear distinction was found between all fermented jujube juices. Furthermore, jujube juice fermented by *L. plantarum* (compared to juice fermented by *L. casei* and juice fermented by *E. faecium*) was found to produce less organic sulphur compounds and more aromatic compounds. In addition, its taste is perceived as less bitter and astringent, resulting in better consumer acceptability.
Zhao et al. (2019) [138]	Tomato juice	Investigating differences in the flavour profiles of tomato juices.	The response of the e-nose sensor set to volatile compounds showed that there was a noticeable change in taste after heat treatment.
Wahia et al. (2020) [130]	Orange juice	Determination of the nutritional properties and microbiological quality of juice during storage.	Based on linear discriminant analysis (LDA), the e-nose sensors were able to distinguish between the aroma of untreated and treated orange juice. It was shown that terpenes, alcohol, and partially aromatic compounds were the main indicators of juice spoilage during storage. It was concluded that the combination of e-nose with gas chromatography coupled to mass spectrometry (GC/MS) could be used as an alternative tool for the rapid detection of the microorganisms responsible for juice spoilage during storage.
Rasekh, Karami (2021) [139]	Two types of natural and industrial juices	Analysis of nine metal oxide semiconductor (MOS) response patterns using artificial neural networks (ANNs) to construct a method for selecting the minimum and most effective number of MOS gas sensors to construct an electronic nose system for the olfactory quality control of various food products.	The method can be used to select the minimum and most efficient number of MOS gas sensors to construct an electronic nose system. It was further found that the use of a minimum number of sensors reduces the cost of building an e-nose, reduces the amount of data input to the processor, and consequently increases the classification accuracy. Hence, the e-nose, in combination with an ANN, can be a highly efficient tool for the rapid and non-destructive classification of fresh and industrial fruit juices.
Nomura et al. (2021) [140]	Banana juice	Measuring and varying the aroma of banana juice from different brands to identify the characteristics, time-related changes, and differences between them.	The FF-2A e-nose used in the study was able to identify the odour of each banana juice in each shop, as well as time-related changes. In addition, it was found that by combining the e-nose with GC-MS, it was possible to assess the components of the odour that changed over time.
Lan et al. (2021) [141]	Fresh mango juice	Measuring the shelf life of non-industrial fresh mango juice.	It was found that the combination of electronic nose and GC-MS could enable effective differentiation and identification of changes in volatile compounds of fresh mango juices under different storage conditions.
Wang et al. (2021) [142]	Noni (*Morinda citrifolia* L.) juice	Evaluation of differences in volatile matter profiles between samples.	The results suggest that the fermentation process may result in the formation of aromatic compounds and that the fermentation treatment may lead to a reduction in sulphides. It was further found that the difference in nitric oxide, alkanes, alcohols, and aromatic and sulphur compounds contributed to the differentiation of Noni juice produced under different fermentation time and scale conditions.
Zhang et al. (2022) [143]	Jujube juice	Analysis of juice aroma; identification of jujube juice aroma differences during fermentation and storage.	It was found that after fermentation and storage, the contents of methane, alcohols, aromatic compounds, terpenes, and other aromatic substances in jujube juice increased. Furthermore, it was shown that the contents of EH (enzymatic hydrolysis) jujube juice were higher than that of NEH (non-enzymatic hydrolysis) jujube juice. Hence, EH treatment could improve the content of flavour components in fermented jujube juice.
Liu et al. (2022) [144]	Tomato juice	Identification and control of cooked flavour in heat-treated tomato juice.	It was found that dimethyl sulphide, dimethyl trisulphide, metional, and 1-octen-3-one were responsible for the cooked off-flavour in tomato juice. It further proved that dimethyl sulphide could be used as a marker compound for the cooked off-flavour in tomato juice.
Wang et al. (2023) [145]	Cherry juice	Identification and analysis of the aroma profile.	The use of e-nose was found to effectively differentiate non-fermented sweet cherry juice and the sweet cherry juice separately inoculated with different LAB strains—Lactic Acid Bacteria.
Bao et al. (2023) [146]	Black carrot juice	Determination of the aroma profile of juice; evaluation of the effect of different sterilisation methods on the quality of black carrot juice.	It was found that the volatiles in black carrot juice were dominated by methane, organic sulphur compounds, terpenes, alcohols, and nitrogen oxides. It was further shown that two of the sterilisation methods ultra-high temperature instantaneous (UHT) and thermal pasteurisation (TP) did not alter the overall odour profile of BCJ.
Zhang et al. (2023) [132]	Strawberry juice	Flavour of freshly squeezed strawberry juice during storage at 4 ± 1 °C.	Using HS-SPME-GC-MS, 17 and 21 volatile organic compounds (VOCs) were detected in the freshly squeezed strawberry juice, while 50 VOCs, including esters, aldehydes, ketones, and alcohols, were detected using GC-IMS.
Zhang et al. (2023) [147]	Strawberry juice	Predicting the increase in total bacterial count in freshly squeezed strawberry juice during cold storage.	It was found that a single electronic nose (E-nose) or electronic tongue (E-tongue) sensor system could be used to predict the total bacterial count in freshly squeezed strawberry juice during cold storage, however, the best accuracy of matching was achieved by combining the two systems.

**Table 3:** The main advantages and disadvantages of the electronic tongue [29,114,151,157,158,163,168,176].

Advantages		Disadvantages	
Advantage	Brief Explanation	Disadvantage	Brief Explanation
Objectivity [177]	Electronic tongues provide objective and consistent measurements, eliminating the subjective influences that can occur with a human taster [177].	Limited complexity [29].	Electronic tongues are simplified analogues of the human senses, meaning that they may not be able to discriminate between such complex taste patterns as humans do [29].
Repeatability [157]	E-tongues provide high repeatability of results, which is crucial for scientific research and quality control in the industry [157].	High initial costs [177].	The cost of purchasing and implementing an electronic tongue can be high, which can be a barrier for smaller businesses or institutions [177].
Speed of analysis [157]	Electronic tongues can carry out analyses in a short time, allowing for problems in production or research to be detected quickly [157].	Difficulty in imitating combinations of sensations [163].	E-tongues can struggle to discriminate and mimic the combination of taste sensations, smell experiences, and responses from visual and touch receptors that the human senses can perceive [163].
Sensitivity to small amounts of substances [163]	E-tongues have the ability to detect small amounts of chemical compounds, even allowing for low-concentration substances to be analysed [163].	Requirement for calibration and maintenance [29].	Electronic tongues require regular calibration and maintenance, which can generate additional costs and labour [29].
No ethical restrictions [168]	The use of e-tongues eliminates the ethical issues associated with human or animal testing [168].	Potential measurement errors [114].	E-tongues can be vulnerable to measurement errors caused by external disturbances such as temperature, humidity, or the presence of other chemicals [114].
Strength [163]	E-tongues are less susceptible to fatigue compared to human testers, allowing for long-term testing without losing the quality of the results [163].	Technical limitations [151].	E-tongues are based on available sensor technologies and data analysis methods, which may limit their ability to recognise non-familiar or new substances [151].
Flexibility [177]	Electronic tongues can be adapted to different applications by using appropriate sensors and analysis methods [177].	Complex software and data analysis [177].	Electronic tongues can require advanced software and analytical skills, which can be challenging for users without adequate training [177].
Safety [177]	E-tongues can analyse inedible, toxic, or dangerous substances, allowing testing under conditions where human testing would be impossible or dangerous [177].	Small number of devices available [157].	There are relatively few devices available on the market for the analysis of liquid samples compared to gas samples, which can make it difficult to choose the right device [157].
Integration with other technologies [176]	Electronic tongues can be easily integrated with other measurement or analysis systems, allowing for more comprehensive studies [176].	Small-scale applications in certain areas [29].	E-tongues may be of limited use in some areas where human senses are more important, such as in assessing the quality of wine or perfume products [29].
Cost reduction [29]	E-tongues reduce the costs associated with testing, eliminating the need to pay human testers or maintain laboratory animals [29].	Lack of a universal standard [158].	The lack of a uniform standard among e-tongues can make it difficult to compare measurement results between different devices and studies [158].

**Table 4:** Examples of studies carried out in the last five years using the e-tongue to evalute the quality of fruit and vegetable juices.

Authors, Publication Year	Type of Juice Analysed	Object of Research	Selected Results
Yu et al. (2018) [192]	Chinese bayberry juice	Evaluation of the taste properties and flavour profile of the juice.	E-tongue was used to identify and characterise the flavour profiles of bayberry juice. Significant differences in sweet and bitter taste were observed between the four types of berry juice. It was further shown that total polyphenols, quinic acid, maleic acid, fructose, citric acid, lactic acid, succinic acid, and sucrose made significant contributions to the flavour characteristics of Chinese bayberry juice (the total polyphenols, quinic acid, maleic acid, fructose, citric acid, lactic acid, succinic acid and sucrose made significant contributions to the taste characteristics of the Chinese bayberry juice).
Daikuzono et al. (2019) [193]	Apple juice	Identifying and differentiating between different types of sugars and juice brands.	The e-language was able to distinguish between different types of sugars (e.g., glucose, fructose, and sucrose), as well as differentiating between commercial brands of apple juice.
Wang, Sun (2019) [191]	Apple juice	Analysis of the feasibility of using an electronic tongue in combination with chemometric analysis for the early detection of *Zygosaccharomyces rouxii* spoilage in apple juice, using the evaluation of taste of the panellists as a reference.	It appeared that the identification of the contaminated juice was completed after 12 h, which is equivalent to the yeast population of less than 2.0 lg colony forming units/mL (identification of the contaminated juice was fulfilled after 12 h, equivalent to the yeast population of less than 2.0 lg colony forming units/mL). At this level, the panellists were unable to discern spoilage. It was therefore concluded that e-tongue appears to be an efficient, rapid, reliable, and cost-effective means of early detection of apple juice spoilage caused by *Z. rouxii*
Gou et al. (2019) [194]	NFC apple juice	Analysis of the effects of different temperatures (room temperature 25 °C, refrigerator temperature 4 °C, freezing temperature −1.5 °C and freezing temperature −18 °C), on the quality and taste qualities of NFC apple juices over a long storage period (150 days).	It was shown that juices stored at different temperatures had a significant difference in flavour, however juice stored at −1.5 °C showed the greatest similarity to freshly squeezed apple juice.
Benjamin, Gamrasni (2020) [195]	Pomegranate juice	Investigating the effect of high-pressure homogenization (HPH) on the microbiological, nutritional, and organoleptic properties of pomegranate juice for the identification of sample aroma.	Analysis of the effects of heat treatment and HPH processes on the taste of pomegranate juice showed similar taste profiles to fresh juice. However, the taste profile of HPH-treated juice was closer to that of fresh juice than that of heat-treated juice. Furthermore, e-language showed positive correlations with the perception of bitterness and astringency for different pomegranate fruit varieties. Additionally, a reduction in the intensity and type of volatile compounds was found in pomegranate juice after pasteurisation at 90 °C for 10 s compared to fresh juice.
Kovacs et al. (2020) [196]	Apple juice	Evaluation of the interfering effects of selected factors (temperature, memory effect, and cross contamination) on sensor signals during e-language measurement and development of drift correction techniques.	The study confirmed that temperature, cross-contamination, and memory effects affect sensor signals. Three drift correction methods were therefore developed and it was shown that the results could be explored for long-term measurements with the e-tongue.
Vitalis et al. (2021) [197]	Plum juice	Detection of *Monilinia* spp. contamination in plum juice and quantitative evaluation of this fungal contamination in raw plum juices.	The e-language was able to distinguish between treated groups, but the classification accuracy was higher for samples stored at 24 °C. E-language was found to be an effective method to reduce food waste by quickly determining fruit quality.
Tan et al. (2021) [198]	Apple/berry juice	Analysis of the influence of the proportion of raw materials used on the sensory quality and antioxidant activity of apple and berry juices.	The blended apple and berry juice was found to have less acidity, astringency and bitterness and improved sensory acceptance. It was also shown that the blended juice could effectively neutralise the flavours of the component juices.
Pardo et al. (2021) [199]	Juice of ‘Aliza’, a new pomelo and mandarin hybrid	Analysis of the sensory properties of Aliza juice (objective evaluation of the taste profile) and their comparison with orange and red grapefruit juices.	E-lingual tests were carried out using sensors for sweetness, sourness, bitterness, saltiness, umami, and astringency. Significant differences in sourness, bitterness, and umami taste were found between the juices analysed: “Aliza”, orange, and grapefruit. Grapefruit juice was found to be significantly more acidic than ‘Aliza’ juice, while orange juice was less acidic than ‘Aliza’ juice. Grapefruit juice was also significantly more bitter than ‘Aliza’ juice; while orange juice was less bitter than ‘Aliza’ juice. In summary, it was found that all three analysed juices had different taste profiles, with the taste of ‘Aliza’ juice being in the middle of the range between orange and grapefruit juices.
Zhang et al. (2022) [147]	Strawberry juice	Predicting the increase in total bacterial count in freshly squeezed strawberry juice during cold storage.	In the study, sweetness was chosen to simulate the growth curve of the total bacterial count. It was found that it was possible to predict bacterial growth in freshly squeezed strawberry juice using e-tongue sensors. However, higher predictive accuracy of fruit juice microbiology could be achieved by combining two sensor systems: e-tongue and e-nose.
Wójcik et al. (2022) [200]	Tomato juice	Testing the effectiveness of e-tongue profiling with innovative electrodes.	Tomato juice available in the local shop in two variants, natural and spicy, was used for the study. In addition, controlled additions of various undesirable substances were added to it. Five profiles of each juice were recorded on all electrode variants, allowing the use of the voltammetric signal from the new electrodes to be tested.
Li et al. (2022) [201]	Yunnan Phyllanthus emblica juice	Determination of nutrients and sensory characteristics of home-made raw *Yunnan phyllanthus* emblica juice, home-made compound *Phyllanthus emblica* juice, and commercial olive drinks.	The results showed that an electronic tongue can distinguish the taste quality of different olive juices, and the combination of this multisensor system with multivariate statistical analysis and sensory evaluation can provide an excellent reference for optimising the formulation of *Phyllanthus emblica* juice
Liu et al. (2023) [202]	Melon juice	Analysis of the effects of ultrasonic (US) and ultra-high pressure (UHP) on the taste qualities of melon juice	The sensors in the e-nose individually represented the intensity of sour, salty, fresh, bitter, tart, and sweet. US was shown to be significantly better at retaining melon juice flavour than UHP.

## Data Availability

Not applicable.
